# Sleep and Organizational Behavior: Implications for Workplace Productivity and Safety

**DOI:** 10.3389/fpsyg.2020.00045

**Published:** 2020-01-31

**Authors:** June J. Pilcher, Drew M. Morris

**Affiliations:** Department of Psychology, Clemson University, Clemson, SC, United States

**Keywords:** sleep, biological rhythm, sleep loss, individual performance, organizational behavior, occupational health

## Abstract

The interaction between sleep and work-related behaviors influence many aspects of employee performance, safety, and health as well as organizational-level success. Although it is well established that quantity and quality of sleep can affect different types of task performance and personal health, the interactions between sleep habits and organizational behaviors have received much less attention. It is important to examine how sleep habits and workplace behaviors relate and the role of the underlying circadian rhythm on the potential impact of sleep and sleepiness in the workplace. Developing a deeper understanding of how sleep habits and sleepiness impact workers and the organization can help provide the necessary background for human resource management to develop more progressive support networks for employees that benefit both the worker and the organization. Human resources and employees should emphasize the impact of good sleep and sleep habits on organizational and individual productivity and safety.

## Introduction

Sleep is an influential component of human health and effective daily functioning and yet is often undervalued in many organizations. Sleep impacts many aspects of employee’s work performance including the ability to adequately respond to rapidly changing work demands and stress-inducing environments and interactions. In the short-term and perhaps at the most basic level, sleep makes us less sleepy and more alert. Poor or inadequate sleep also has a negative impact on many longer-term factors relevant to organizational behavior and personal health including self-control and decision making (Hagger, 2014; Pilcher et al., 2015b), subjective effort (Engle-Friedman and Riela, 2004), immunosuppression (Irwin, 2015), and a variety of performance measures (Lim and Dinges, 2010). Furthermore, our endogenous circadian rhythms impact not only our sleep-wake cycle by encouraging us to sleep at night but also affect our daytime alertness and performance. The organization can also have an impact on our endogenous need for sleep and our circadian rhythms. When organizations create an environment that increases work requirements, this creates additional stress beyond simply the work demands for the employee as well as the employee’s family including poor sleep and other social and health concerns (Mariappanadar, 2014). In addition, many organizational demands, such as shiftwork and travel, challenge our circadian rhythms and create a sustained physiological drive for sleep. As such, sleep and circadian rhythms and their relationship to employee productivity, safety, and health are important concerns for human resource management.

## Sleep and Circadian Rhythms

Sleep is often described by two main components, sleep quantity and sleep quality. Sleep quantity is the amount of time spent asleep each night while sleep quality reflects features of sleep related to how well the person slept, such as time taken to fall asleep, number of awakenings during sleep, and how well rested the individual feels after waking (Pilcher et al., 1997). Both sleep quantity and sleep quality are important when considering how sleep impacts daily functioning, and can independently vary based on sleep habits and the presence of health issues or clinical sleep disorders. A recent meta-analysis found that sleep quality was better related than sleep quantity with employee perceptions and emotions such as workload, perceived control, and general strain (Litwiller et al., 2017). Decreased sleep quality is also associated with difficulties with social interactions in the workplace including feelings of ostracism (Chen and Li, 2019). In addition, work schedules can require the person to be awake and functioning very early in the morning, often resulting in decreased sleep quantity for the employee. Or persons with sleep disorders, such as sleep apnea, may have a schedule that allows them to sleep for 7–8 h, but their condition causes them to wake up regularly throughout the night, leading to decreased sleep quantity and poor sleep quality (Lopez et al., 2013). It is important to note that both decreased sleep quantity and poor sleep quality will increase sleepiness during work hours and can negatively impact the worker’s performance and health.

The physiological need for sleep resulting in a daily sleep–wake cycle is a part of and interacts with our endogenous circadian rhythm. Circadian rhythms are physiological and behavioral cycles that occur on approximately a 24-h basis. In terms of our sleep–wake cycle, the desire to sleep is generated as a part of the circadian rhythm to sleep at night as well as a physiological need for sleep that generates roughly at the rate of 1 h of sleep for every 2 h awake. As such, each day we gradually accumulate the need to sleep as a result of being awake but are also influenced by the circadian drive for sleep. Because our daily rhythms are synchronized with the environment to promote sleepiness at the appropriate time, the circadian rhythm acts as an internal clock, and is influenced by bright light exposure, eating, and physical activity. When occupational needs require individuals to shift their regular sleep–wake routine, whether due to shift work scheduling or traveling across time zones, the inertia of the endogenous circadian rhythm can promote sleepiness or wakefulness at inappropriate times leading to occupational health and safety risks.

Another challenge to the endogenous circadian rhythm occurs in workers in the circumpolar regions where sun light exposure and temperature change dramatically due to seasonal variation which also impact sleep habits and can negatively affect alertness and performance (Lan et al., 2017; Morris et al., 2017b). For example, seasonal affective disorder can occur in individuals exposed to extreme changes in environmental conditions, such as dramatic changes in sun light exposure (Rosenthal et al., 1984). Persons living in regions with limited sun light exposure in the winter time can experience seasonal affective disorder where periods of depression occur during the winter months but then gradually decrease as the day length increases. In fact, day length is related to use of antidepressants in United Kingdom (Lansdall-Welfare et al., 2019). These work-related challenges to the circadian system are common occurrences in many work settings and require additional attention when considering the impact of sleep on organizational behaviors and occupational health issues.

## Assessment of Sleep and Sleepiness

Sleep is both a physiological event and a personal experience. As such, methods for measuring sleep and sleepiness incorporate both objective and subjective measures. Laboratory measures of sleep include physiological methods such as polysomnography, which includes measures such as eye tracking, brain and muscle activity, respiration, oxygen levels, and cardiac activity to precisely monitor wakefulness and sleep. Although these measures are widely used in clinical settings, there are limitations to standard sleep stage scoring (Pilcher and Schulz, 1987; Watanabe and Watanabe, 2004). Physiological measures of sleepiness can include using polysomnography to monitor how long an individual may take to either fall asleep (multiple sleep latency test; Carskadon and Dement, 1982) or remain awake (maintenance of wakefulness test; Mitler et al., 1982) when in a resting position. Either of these measures can provide an objective estimate of sleepiness when the person should be awake. These physiological methods provide sleep scientists and clinicians the ability to track the onset of sleep, monitor sleep stages, assess physiological sleepiness, and diagnose sleep disorders. However, because of the invasiveness and cost of physiological equipment, this technology is seldom applied in an occupational setting to measure sleep or sleepiness. One method that can help address this issue is actigraphy where portable activity monitors are used to provide an estimate of sleep time in the individual. Although actigraphy is not as accurate as polysomnography, it does provide a useful estimate of sleep–wake time that can be compared within individuals across nights.

Many studies, particularly those in the workplace, ask individuals to record characteristics of their nightly sleep in a sleep log. Individuals record relevant information before and after a night of sleep, including when they believe themselves to have fallen asleep, how long they slept, and how often they awoke during the night (Carney et al., 2012). Scales such as the Pittsburgh Sleep Quality Index ask additional questions about sleep medication use and patterns of behavior to quantify sleep habits (Buysse et al., 1989). These types of subjective measures provide information about sleep habits but are dependent upon the individual’s assessment of their own sleep patterns. In recent years, wearable technology has been used to supplement self-report information, thus providing an objective means of monitoring sleep and sleep habits. Wearable technology allows individuals to track their sleep patterns with relatively little effort. These wearable devices range in price and complexity, but even inexpensive step trackers can use motion tracking to estimate time asleep. Although consumer technology seems to overestimate total sleep time and underestimate sleep disruptions (Kolla et al., 2016), they can provide a meaningful measure of sleep habits when comparing across time within an individual.

Another metric of interest to organizations is the measure of daytime sleepiness. For many researchers and clinicians much of the purpose of tracking nighttime sleep is to better predict daytime fatigue and sleepiness in terms of performance and well-being as well as to estimate the likelihood of falling asleep during the work day. Excessive daytime sleepiness is directly related to risk of at-work injuries, task mistakes, and long-term negative health outcomes (Pagel, 2009). Sleepiness surveys such as the Stanford Sleepiness Scale, that asks individuals to describe their current feeling of sleepiness (Hoddes et al., 1973), and the Epworth Sleepiness Scale, that asks individuals their likelihood of falling asleep given certain scenarios (Johns, 1991) are used regularly in research and clinical settings. Although these sleepiness measures have some limitations (Shahid et al., 2010; Pilcher et al., 2018), both scales can be administered in less than 2 min, making them practical for a workplace setting.

Sleep-related fatigue can also be quantified using performance metrics and non-invasive physiological indices. For example, both simple reaction time on a psychomotor vigilance task and eye movement speed slow proportionately with the severity of sleep deprivation, while instances of attention lapses increase proportionately (Morris et al., 2015). If performance is already being tracked as part of normal job function or can be added as an additional simple task, using a performance metric offers an opportunity to assess the potential detrimental effects of sleepiness with little interruption to the workplace. In addition, less-invasive physiological indices of the circadian rhythm, such as body temperature, show promise in predicting workplace error due to sleepiness (Morris et al., 2017a). When considering potential concerns in occupational health and safety, work environments that regularly challenge the circadian system through shift work, regular travel across time zones, or work in diverse global regions could be designed to assess performance or physiological indices to monitor potential safety and health issues due to sleep disturbances and sleepiness.

## Sleep and the Workplace

Human resource management includes supervision of risk factors that impact employee health and well-being as well as productivity in the workplace (Becker and Smidt, 2016). The negative impact of sleep deprivation is one area that is often undervalued by workers and by human resource management. Sleep deprivation resulting either from poor choices related to sleep habits or to occupational requirements such as shift work is a common cause of sleep- and sleepiness-related detriments in the workplace. Sleep deprivation negatively impacts a wide range of employee performance, health, and well-being issues including immune defense reaction (Majde and Krueger, 2005), cardiovascular functioning (Walker et al., 2009), metabolic disorders (Kecklund and Axelsson, 2016), mood disorders (Touitou et al., 2017); affective reactivity (Pilcher et al., 2015a), motivation (Odle-Dusseau et al., 2010), subjective effort (Engle-Friedman and Riela, 2004), accidents in the workplace (Uehli et al., 2014), and performance on many types of vigilance and more complex cognitive tasks (Pilcher et al., 2007; Pilcher et al., 2016). In addition, daytime sleepiness is related to higher mortality rates (Empana et al., 2009), cardiovascular disease and diabetes (Chasens et al., 2009), and fatigue-related accidents (Melamed and Oksenberg, 2002). This wide range of effects related to poor and inadequate sleep has led some to declare a sleep crisis as a public health issue (Barnes and Drake, 2015).

### Shift Work

Because of the prevalence of sleep deprivation and daytime sleepiness, health agencies in many countries, such as the US Centers for Disease Control and Prevention, are increasingly monitoring population-based sleep habits (Gelaye et al., 2014). Irregular work hours, such as those seen in shift work schedules, have a negative impact on work performance and can continue over days off (Åkerstedt, 2003). Data collected by the National Center for Health Statistics indicate that 29.9% of working adults average less than 6 h of sleep a night (Luckhaupt et al., 2010). Among specific industries, the highest percentage of individuals experiencing sleep loss were those involved in the management of companies and enterprises (40.5%). Manufacturing and transportation follow closely as the second and third highest industries for sleep loss respectively, largely due to the prevalence of shift work scheduling and work demands across the 24-h day. These patterns of sleep disturbances occur in multiple societies (Åkerstedt, 1998; Dregan and Armstrong, 2011). Furthermore, between 15 and 30 percent of the working population in developed countries are on shift work schedules, often working against the body’s endogenous circadian rhythm and, as such, promoting sleep disturbances and sleep disorders (Boivin and Boudreau, 2014).

Shift work-related sleep disturbances are more common when occupations require workers to sleep during the day, a common result of working at night (Drake et al., 2004). Daytime sleep is challenging for most workers due to a variety of issues such as family demands, environmental light and noise, and the natural circadian pressure to be awake during the day. Some studies suggest that sleep quantity and sleep efficiency are lower in night workers who must sleep during the day compared to rotating shift workers who sleep at different times across the 24-h day depending on their shift work schedule (Drake et al., 2004). However, this may depend on the pattern of night shifts a worker experiences or even individual differences. There is some evidence that permanent night shifts can result in a more stable sleep pattern for many workers than rotating shifts (Pilcher et al., 2000). It is important to note; however, that night shift workers will always have to combat the natural circadian rhythm to be asleep at night and awake during the day. Perhaps the best way for night shift workers to adapt to working at night is to maintain the pattern of being awake at night and sleeping during the day on their days off. Even then, the presence of sunlight during the day will bolster the circadian pressure to be awake during the day and to sleep at night. Although there is some debate among sleep scientists about the relative merits of different shift work schedules, it is clear that night shift work will result in sleep difficulties and, almost always, in sleep loss. The answer is to not require persons to be alert and work at night when their circadian rhythm is encouraging sleepiness and sleep. However, since 24-h-a-day operations are necessary in most societies, the best option is to develop health promotion paradigms and countermeasures to decrease the negative side effects of night shift work.

The need for 24-h-a-day operations in developed countries has increased the likelihood that workers will experience fatigue, sleepiness, and decreased performance skills as part of their daily lives (Åkerstedt, 2007; Arendt, 2010). Evidence also suggests that the more one works, the less time the person sleeps, even on days off (Basner et al., 2007; Krueger and Friedman, 2009). It is important to note; however, that the short-term and long-term impact of sleep deprivation and changes in sleep patterns due to occupational demands vary according to specific characteristics of the work requirements as well as the worker.

### Individual Differences

Although most organizational research assumes a degree of homogeneity to the working population, individual differences can affect how the working environment impacts the employee. Factors such as gender and age contribute to differences in sleep quantity and quality in addition to general well-being in workers. Studies of employee health and well-being have shown that women shift workers have an increased risk of poor sleep quantity and quality compared to their male counterparts (Chung et al., 2009). This includes difficulty falling asleep, staying asleep, and a higher likelihood to use sleep medications to compensate for poor sleep (Marquie and Foret, 1999). Women also suffer more from work-family imbalance issues than men. Social and family obligations may partially account for the observed gender difference in that women often are responsible for more of the family and home duties than males; however, gender differences in the circadian rhythm may also contribute to the difference seen between females and males (Paschos and FitzGerald, 2017). For example, even in identical environments, the female circadian clock tends to be set earlier and slightly shorter on average than the male circadian clock (Duffy et al., 2011). In addition, growing evidence suggests that many of the differences in sleep between females and males could be related to the lower socioeconomic status of females in comparison to males (Arber et al., 2009). These differences in sleep patterns between females and males can contribute to different responses from females when meeting work demands that challenge the circadian rhythm.

Age is also a primary predictor of sleep and behavior, an issue that has become increasingly important as the average workforce age increases (Ng and Feldman, 2010). Although the time spent in bed remains relatively stable throughout the working adult years, there are several factors that contribute to poorer sleep in later adulthood. As early as the middle twenties, sleep patterns begin to shift and continue to change throughout adulthood and into retirement age (Ohayon et al., 2004). With age comes a decrease in the amount of deep sleep, generally associated with poorer cognitive performance, and an increase in the number of awakenings during the night, which is linked to less sleep quantity and poorer sleep quality (Crowley, 2011). These changes in sleep in adulthood can have a negative effect on individual health, decreased cognitive and physical ability during the work day, and a higher risk of injury. Although we do not yet fully understand these age-related changes in sleep, one contributing factor is an age-related decline in the endogenous circadian rhythm output which may help destabilize the sleep-wake cycle (Nakamura et al., 2011). However, it is important to note that lifestyle habits can also play a role in how individuals adapt to the potential detrimental effects of working conditions that challenge the sleep-wake cycle. For example, one study in nurses found the highest prevalence of excessive daytime sleepiness (29.3%) in those 20–29 years of age, compared to those in their 30 s (24.7%), 40 s (15.5%), or 50 s (12.3%), largely due to social choices such as maintaining or not maintaining regular sleep schedules (Suzuki et al., 2005).

Performance in the workplace can also be moderated by the endogenous circadian rhythm. The endogenous circadian rhythm in a few individuals can be slightly shorter than 24 h but averages 24.2 h in length (Czeisler et al., 1999). There is evidence that this variation in the circadian rhythm can be used to define individuals as morning types who have shorter circadian periods or as evening types who have longer circadian periods, with most individuals somewhere between the two extremes (Duffy et al., 2001). A convenient method to determine a person’s chronotype is through self-report questionnaires (Horne and Östberg, 1976) which can be easily administered in a variety of settings. For example, multi-country data gathered through a self-report survey suggest that chronotype affects sleep duration and itself is dependent on age and sex (Roenneberg et al., 2007).

Individuals who do fall in the extremes of morningness and eveningness can experience difficulties with on-the-job performance and functioning. Those with a disposition toward morningness are found to be more productive during the earlier hours of the day, while those who identify as evening types tend to be more productive during the later portion of the day (Preckel et al., 2011). In addition, the chronotypes follow age-related patterns with early career individuals in their 20 s tending toward eveningness and later career individuals tending toward morningness (Monk and Kupfer, 2007; Preckel et al., 2011). The morningness versus eveningness of an individual also impacts sleep and social activities. Circadian rhythms contribute to less sleep quantity and poorer sleep quality in persons with eveningness which will in turn result in performance degradation in the workplace as discussed previously. In a social context, eveningness can negatively impact social interactions and contribute to feelings of stress in the earlier part of the work day (Mecacci and Rocchetti, 1998; Cofer et al., 1999). Furthermore, changes in exposure to sunlight due to where a person lives within a given time zone can affect the circadian clock. It has been shown that persons living on the eastern edge of a time zone tend to be more likely to be morning people while persons in subtropical regions tend toward eveningness compared to those who live in more northern regions (Randler, 2008). It is also interesting to note that chronotypes are influenced by how the pressure or need to sleep is dissipated by sleeping (Mongrain et al., 2006), thus adding to the complexity of workers better managing their sleep to improve their work-related performance.

Race and cross-cultural differences have also emerged as relevant factors when considering employee sleep and health (Grandner et al., 2016). A meta-analysis concluded that African Americans slept less than white Americans each night and had less deep sleep overall (Ruiter et al., 2011). Moreover, minority groups, in general, sleep less than non-minorities (Jean-Louis et al., 2000), while African/Caribbean immigrants sleep less than white Americans even after controlling for education and occupation (Ertel et al., 2011). Sleep quantity; however, is not the only potential concern when examining the effects of sleep on organizational behaviors and occupational health issues. For example, sleep quality is better related to health and well-being than sleep quantity in persons sleeping about 7 h a night (Pilcher et al., 1997). Sleep quality also seems to be a mitigating factor when examining the effects of socioeconomic status, race, and sleep on health (Moore et al., 2002) suggesting that both lower socioeconomic status and poor sleep negatively affect health in minority groups. Measures related to sleep also vary across different countries and cultures. Germans, for example, differ in their sleep-wake patterns and chronotypes from Indians and Slovakians (Randler et al., 2015) while sleep problems related to aging relates to increased health and well-being issues in multiple countries across Africa and Asia (Stranges et al., 2012).

A variety of other factors impact the ability of individuals to maintain a healthy sleep–wake cycle. Persons suffering from clinical sleep disorders, such as insomnia and sleep apnea, have more work impairments (Swanson et al., 2011) and are more likely to have substance use disorders than normal sleepers (Fortuna et al., 2018). The presence of sleep disorders has also been used to predict risk of substance use in the future (Wong et al., 2004). Research suggests there is a bidirectional relationship between sleep and substance use that can decrease occupational productivity (Mullins et al., 2014). In addition, cultural standards influence sleep. Social demands such as school or work start times alter the sleep–wake cycle (Jenni and O’Connor, 2005). Personal decisions for sleep time within a family such as earlier or later bedtimes for children can change the sleep habits of the parents and negatively affect their work performance the next day (Giannotti and Cortesi, 2009). Finally, cultural expectations for sleep habits can impact sleep and work performance and can range widely across cultures both for sleeping at night and daytime napping which can impact on-the-job performance. As we have seen, decreased sleeping at night negatively impacts work performance. In contrast, daytime napping can help mitigate this effect and potentially improve performance after the nap; however, this effect varies across individuals.

### Work Performance

Poor and inadequate sleep results in a variety of cognitive deficits, including an inability to maintain attention, decreased alertness, delayed reaction time, dulled auditory and visual perception, altered emotional processing, and a general inability to think clearly (Lim and Dinges, 2010). When considering sleep-deprived workers, this results in a decrease in job-related performance and a propensity for errors. For example, many studies have found that sleep deprivation and sleepiness result in an increased likelihood of medical-related errors. Excessive daytime sleepiness has been found to predict drug administration errors and incorrect operation of medical equipment (Suzuki et al., 2005). In addition, nurses alter their medical-related decision-making across a 12-h shift (McClelland et al., 2013) and on-call scheduling results in performance decrements in physicians (Pitkanen et al., 2008).

Sleep loss can also promote injury both during work and outside of work hours. Employees at four United States corporations were surveyed about sleep habits and those reporting insufficient sleep were nearly twice as likely to unintentionally sleep during work, fall asleep while driving, and injure themselves at home due to sleepiness compared to good sleepers (Rosekind et al., 2010). Workers suffering from obstructive sleep apnea and the resulting sleep loss and daytime sleepiness are twice as likely to injure themselves while at work than those without sleep apnea (Garbarino et al., 2016; Hirsch et al., 2016). Furthermore, in a study of 160 fatal occupational accidents, sleep difficulties were more predictive of fatal accidents than how physically strenuous the work was, how hectic the work was, the age of the individual, or whether the individual was working overtime (Åkerstedt et al., 2002). More specifically, risk of occupational injury due to sleepiness is particularly high in the transportation industry. In one sample, one-in-four professional drivers were affected by insomnia, which doubled their risk of crashing while driving for work and tripled their risk of having a near-miss accident compared to non-insomniac drivers (Garbarino et al., 2017).

As noted earlier, sleep deprivation negatively impacts motivation to perform well when working. It is important to understand how sustained performance under sleep loss conditions impacts subjective perceptions as those could be the first indicator of a potential negative reaction by the individual (Jones et al., 2006). It is well documented that sleep deprivation negatively impacts mood and increases sleepiness (Pilcher and Huffcutt, 1996; Driesen et al., 2010). The type of task could also affect how the worker reacts when sleep deprived with vigilance tasks being more negatively affected than more complex tasks (Odle-Dusseau et al., 2010). The desynchronization of the sleep-wake cycle as well as the loss of sleep as seen in many occupations can be a direct cause of stress in individuals (Jaffe et al., 1996). Furthermore, Kucharczyk et al. (2012) found that persons suffering from insomnia are more likely to struggle with professional development in general, resulting in a lower likelihood of job promotion, and a higher probability of dismissal. Although less research has focused on the individual’s perceptions when working under sleep-deprived conditions as well as the broader implications of sleepiness in the workplace, the evidence suggests that these could be important issues for human resource management.

### On-the-Job Behaviors

Sleep plays a unique role in work behaviors due to the impact of sleep loss and sleepiness on workers’ overall situational and emotional processing. van der Helm et al. (2010) showed that sleep deprivation impairs the ability to judge human facial emotion which could disrupt affective social cues valuable to interpersonal communication. In addition, sleep deprivation negatively impacts the individual’s response to positive stimuli (Pilcher et al., 2015a) which could result in more focused responding to perceived negative events. In an occupational setting, this can result in miscommunication, social misjudgment, and tension between coworkers. Barber and Budnick (2015) found that sleepiness during work was related to aggressive behaviors in the workplace. Tired individuals were more likely to rationalize using aggressive behavior and avoiding rules perceived as unfair (Berry et al., 2010). These types of potential issues are of particular concern in that workers are more likely to fully engage in their workplace when they have emotional and cognitive investment in the system (Voronov and Vince, 2012) and a strong work identity (Knez, 2016), something that sleep loss and sleepiness could negatively impact.

Sleep deprivation is also related to employee absenteeism. Non-shift work employees across a variety of blue-collar and clerical occupations who self-report more daytime sleepiness are significantly more likely to take work absences (Philip et al., 2001) and more likely to arrive late or leave early (Swanson et al., 2011). Although absenteeism could be explained by considering the relationship between sleep and illness, other research supports the possibility of willful absenteeism. Barnes et al. (2011) found that poor sleep quantity and quality could predict unethical behaviors in a work setting. In addition, workers with poorer sleep were more likely to lie about their own performance scores and were rated as showing more unethical behavior on an ethics scale by their supervisors (Akaah, 1992). This included actions such as misusing sick days and company time, claiming credit for someone else’s work, and divulging confidential company information.

A variety of factors in and out of the workplace can contribute to sleep loss and sleepiness, including many psychosocial work factors. Social support impacts the ability to react to challenging conditions and exert the self-control needed to successfully navigate many stress-inducing situations (Pilcher and Bryant, 2016). In a sample of Japanese workers, decreased social support and interpersonal conflicts at work were related to an increase in risk of insomnia (Nakata et al., 2004). Similarly, research shows that social exclusion at work increases risk for sleep disturbances (Pereira et al., 2013). These issues likely stem from increased physiological arousal due to stress and anxiety as well as a negative shift in the victim’s sense of self and feelings of support for work mates. Negative events outside of the workplace similarly contribute to sleep loss, commonly through parasomnias such as nightmares in the case of traumatic events (Mysliwiec et al., 2014). Indeed, feelings of stress from daily life, including psychosocial stress but also everyday challenges, predict day to day sleep quality (Åkerstedt et al., 2012) and thus impact on-the-job behaviors.

## Sleep and Health

Many people recognize that maintaining good sleep habits is part of making healthy lifestyle choices; however, it can be surprisingly difficult to maintain good sleep habits which can lead to negative consequences on long-term health. Sleep scientists have found numerous links between sleep and health. Poor sleep over a longer period of time is related to increased risk of cardiovascular disease and diabetes (Chasens et al., 2009) and higher risk of developing dementia and Alzheimer’s disease (Benedict et al., 2015). Studies have also found increased mortality rates associated with either shorter sleep periods (less than 3.5–4.5 h) or longer sleep periods (greater than 8 h) (Kripke et al., 2002) as well as with excessive daytime sleepiness (Empana et al., 2009). Moreover, shift workers have a higher risk factor for developing health issues. One review study concluded that shift workers, particularly night shift workers, experience more severe gastrointestinal, neuro-psychological, and cardiovascular issues than non-shift workers (Costa, 1996). A second review study concluded that shift workers are more likely to suffer from peptic ulcer disease and coronary heart disease (Knutsson, 2003) than non-shift workers. More generally, night shift workers can experience a wide range of long-term issues that can contribute to poor health over time. Nurses working the night shift, for example, have significantly higher incidences of obesity, higher caloric intake and tobacco use, and poorer sleep (Ramin et al., 2015). Finally, shift workers experience a wide range of issues that can negatively affect long-term health including concerns with families and social life, increased fatigue, and increased risk of on-the-job accidents (Harrington, 2001).

There is also increasing evidence that sleep is specifically involved in the functioning of the immune system (Bryant et al., 2004). Research suggests that proper functioning of many components of the immune system such as T-cells and inflammation markers as well as necessary endocrine functioning depend on the timing of sleep and the endogenous circadian rhythm to sleep at night (Lange et al., 2010). More specifically, immunology studies show that sleep loss is associated with increased secretion of proinflammatory cells while curtailing the antiviral immune responses (Irwin, 2015). Other studies also suggest a link between exposure to viruses, poor sleep, and developing an illness. For example, when exposed to the rhinovirus, participants who averaged less than 7 h of sleep were three times more likely to show clinical symptoms of the common cold than those who slept 8 h (Cohen et al., 2009). Cohen and colleagues also found that people with low sleep efficiency were five times more likely to show cold symptoms when exposed to the rhinovirus. Furthermore, just feeling rested after sleeping does not seem to be enough to ensure a properly functioning immune system. Although employees who sleep for fewer hours can report deep sleep and may even feel rested upon awakening, they are still at risk for developing an illness (Vgontzas et al., 2004). Sleep deprivation also appears to attenuate the antibody response to influenza and hepatitis vaccinations (Spiegel et al., 2002). This effect is further compounded with stress, as higher subjective reports of stress with poor sleep further diminishes the humoral immune response to immunization (Miller et al., 2004).

Even relatively short-term sleep loss can have adverse effects on key health indicators. In general, short-term sleep restriction can decrease glucose tolerance, increase blood pressure, increase reactivity of the sympathetic nervous system, decrease leptin levels, and increase immunological markers of inflammation (Alvarez and Ayas, 2004). One night of sleep deprivation results in increased systolic blood pressure in middle-aged adults (Kato et al., 2000) and in elevated diastolic blood pressure in young healthy adults with a family history of hypertension (McCubbin et al., 2010). Partial sleep deprivation also has negative effects on several physiological indicators of health. For example, blood pressure is increased in persons reporting 3.6 h of sleep at night (Tochikubo et al., 1996). Approximately 4 h of sleep at night is also related to impaired glucose reactivity, increased sympathetic response, higher cortisol levels, and reduced leptin levels (Spiegel et al., 1999) as well as increased levels of C-reactive protein, an indicator of inflammation related to heart disease (Meier-Ewert et al., 2004). Both total and partial sleep deprivation have negative effects on many health-related physiological indices suggesting that helping workers prioritize good sleep habits is essential for good health in the workers and long-term organizational success.

## Human Resources Impact

When organizations go beyond the goal of financial gain to embrace corporate social responsibility and support the good of society, employees work harder and better contribute to the effectiveness of the organization. Better integrating the concept of corporate social responsibility with well-being of employees could also have positive effects on the organization (Glavas, 2016). Although many human resource models incorporate necessary functions for organizational success such as hiring and staffing practices, benefits and compensation, and training for employees, it is less common for human resource divisions to focus on employee well-being.

Work-related stress and strains are related to the employee’s perceived well-being but can be compensated for through social support (Giorgi et al., 2017). Human resources could be part of that social support system. Organizations can benefit if human resources create more of a partnership with their workers and include the well-being of workers in the different decision processes that typically take place in human resources. For example, human resources could focus on job design and anti-harassment practices that would benefit the workers and increase performance rates (Guest, 2002).

Sleep loss and sleepiness is one well-being issue that directly impacts the functioning of employees on many levels. Although less research has examined the impact of sleep loss and sleepiness at the organizational level, these are important issues for employees, managers, and human resources. For example, research suggests that changes in behavior due to sleep loss could be considered during personnel selection. Person-specific sensitivity to the negative effects of sleep deprivation has been considered as a trait characteristic (Van Dongen, 2006) that could be used when hiring individuals for shift work. Furthermore, employee selection tools on aggressive behaviors could be used as an indicator of employee responses in work environments associated with sleep loss (Barber and Budnick, 2015). The implications of these findings suggest that human resources could mitigate future personnel problems at the level of hiring or when assigning employees to a new work position by screening for individual responsiveness to sleep loss and sleepiness. The potential benefits of organizations taking these steps are more apparent when considering that individuals do not appear to self-select occupations based on their own ability to cope with sleepiness-inducing work environments or schedules (Van Dongen, 2006).

Research suggests that working conditions can negatively impact employee health (Urtasun and Nuñez, 2018). As such, it is important to consider how human resources can promote healthier work environments. One method is by promoting workplace practices that could curtail on-the-job sleepiness. Caruso and Hitchcock (2010) have suggested that traditional tactics for better managing sleepiness related to shift work, such as providing for nap breaks, are promising countermeasures. Human resources could also consider work scheduling practices that provides flexible working hours for employees where employees could choose either earlier or later work hours to better match their chronotype or to better match their family or other nonwork-related demands. Organizations can also provide adaptation time for workers to adapt to new time zones when traveling. In addition, there is growing evidence for the use of light exposure to promote alertness. Light is connected to the circadian rhythm and sleep–wake cycle and may help establish alertness at appropriate times. Research suggests that allowing for regular exposure to natural sunlight during the day can help ward off daytime sleepiness (Caldwell et al., 2009). In the case of night shift work, brief half-hour exposure to bright artificial light during the night has been shown to reduce subjective sleepiness and encourage alertness (Cajochen et al., 2000; Smolders and de Kort, 2014). Although there is much work yet to do at the organizational level, current research suggests several possible workplace interventions that human resource divisions could explore as potential countermeasures to help employees better manage their on-the-job alertness levels.

## Conclusion and Implications

In recent years, some organizations have started to emphasize better understanding how sleep habits and sleepiness affect their employees and the workplace. Although research has connected poor sleep to decrements in many major cognitive processes as well as negative health outcomes, the literature addressing occupational interventions is still developing. The current summary relating sleep habits and sleep deprivation with organizational behavior (see Table 1 for a summary of the cited literature in this review), will help provide background for new research.

**TABLE 1:** Summary of Literature Cited.

Authors	Title	Year	Major Findings
Akaah, I. P.	Social inclusion as a marketing ethics correlate	1992	In organizations of warmth, marketing professionals show lower ethical behavior. In organizations with strong member identity, marketing professionals show higher ethical behavior.

Åkerstedt, T.	Shift work and disturbed sleep/wakefulness	1998	Sleep disturbances seen in shift workers is similar to clinical insomnia and results in increased fatigue-related accidents and less productivity. Discusses potential counter-measures.

Åkerstedt, T.	Shift work and disturbed sleep/wakefulness	2003	The most common sleep-related difficulties related to shift work include difficulty going to sleep, less sleep, and sleepiness during working hours.
Åkerstedt, T.	Altered sleep/wake patterns and mental performance	2007	Sleep patterns different from sleeping at night is associated with on-the-job mistakes and accidents and increased health risks. However, there is limited research across different industries.

Åkerstedt, T., P. Fredlund, M. Gillberg, B. Jansson	A prospective study of fatal occupational accidents-relationship to sleeping difficulties and occupational factors	2002	Self-reported sleep problems, being male, and working on non-day shifts is associated with accidental death at work.

Åkerstedt, T., N. Orsini, H. Petersen, J. Axelsson, et al.	Predicting sleep quality from stress and prior sleep–a study of day-to-day covariation across six weeks	2012	Sleep quality is related primarily to bedtime stress and worries but is also related to late awakening, short prior sleep, high quality of prior sleep, and good health on the previous day.

Alvarez, G. G., N. T. Avas	The impact of daily sleep duration on health: A review of the literature	2004	Both experimental studies examining short-term sleep loss and epidemiologic studies suggests that shorter sleep duration negatively affects health.

Arber, S., M. Bote, R. Meadows	Gender and socio-economic patterning of self-reported sleep problems in Britain	2009	Sleep quality is related to several facets of socioeconomic status. Sleep difficulties may contribute to the link between low SES and poor health.

Arendt, J.	Shift work: Coping with the biological clock	2010	Shift work results in desynchronization of the internal circadian pacemaker contributing to sleep, performance, and health-related problems. Bright light exposure at night may be particularly problematical.

Barber, L. K., C. J. Budnick	Turning molehills into mountains: Sleepiness increases workplace interpretive bias	2015	Sleepiness increases interpretive bias when the workplace is unfair but not when the workplace is fair. This relationship is unrelated to negative affect, ego depletion, and personality variables.

Barnes, C. M., C. L. Drake	Prioritizing sleep health: Public health policy recommendations	2015	Focuses on developing new public policies on good sleep including later school start times, better regulation of work hours and schedules, eliminating daylight savings time, better awareness of impact of electronic media on sleep, and better in-home testing for sleep disorders.

Barnes, C. M., J. Schaubroeck, M. Huth, S. Ghumman	Lack of sleep and unethical conduct	2011	Decreased sleep quantity and poor sleep quality are positively related to supervisor rated unethical behavior in employees.

Barnes, C. M., D. T. Wagner, S. Ghumman	Borrowing from sleep to pay work and family: Expanding time-based conflict to the broader nonwork domain	2012	Time spent sleeping depends on work time and family time where when work demands and family needs increase, sleep time decreases.

Basner, M., K. M. Fomberstein, F. M. Razavi, S. Banks, et al.	American time use survey: Sleep time and its relationship to waking activities	2007	Sleep time is negatively associated with work time, travel and commuting time, and time socializing.

Becker, K., M. Smidt	A risk perspective on human resource management: A review and directions for future research	2016	Discusses a better merging of human resource management and risk management in organizations. Covers the need to balance organizational returns with the risks to human resources.

Benedict, C., L. Byberg, J. Cedernaes, P. S. Hogenkamp, et al.	Self-reported sleep disturbance is associated with Alzheimer’s disease risk in men	2015	Men reporting sleep disturbances were more likely to develop dementia and Alzheimer’s disease.
Berry, C. M., P. R. Sackett, V. Tobares	A meta-analysis of conditional reasoning tests of aggression	2010	Aggression can used to predict job performance and counterproductive work behavior.

Boivin, D. B., P. Boudreau	Impacts of shift work on sleep and circadian rhythms	2014	Shift work create challenges to sleep habits and circadian patterns in workers. Worker tolerance to shift work depends on many factors that must be addressed to counter the potential impacts of shift work.

Bryant, P. A., J. Trinder, N. Curtis	Sick and tired: Does sleep have a vital role in the immune system?	2004	Sleep deprivation negatively affects immune-cell number, function, and cytokine production. Chronic partial sleep loss may be more detrimental to immune function than short-term total sleep deprivation.

Buysse, D. J., C. F. Reynolds, T. H. Monk, S. R. Berman, D. J. Kupfer	The Pittsburgh Sleep Quality Index: A new instrument for psychiatric practice and research	1989	Reports on initial development of the PSQI using good and poor sleepers. Suggests that PSQI can be used in research and clinical settings.

Cajochen, C., J. M. Zeitzer, C. A. Czeisler, D. Dijk	Dose-response relationship for light intensity and ocular and electroencephalographic correlates of human alertness	2000	Light exposure during early biological night resulted in an acute alerting effect in human electroencephalogram, decrease in melatonin secretion, and decrease in self-reported sleepiness.

Caldwell, J. A., M. M. Mallis, J. L. Caldwell, M. A. Paul, et al.	Fatigue countermeasures in aviation	2009	Recommended countermeasures include better education about dangers of sleepiness and fatigue, better fatigue risk management systems, improved nap scheduling, careful use of alertness-enhancing substances, and improved use of fatigue detection technologies.

Carney, C. E., D. J. Buysse, S. Ancoli-Israel, J. D. Edinger, et al.	The consensus sleep diary: Standardizing prospective sleep self-monitoring	2012	Reports on development of Core sleep diary including questions about time in bed, length of time to fall asleep, and sleep quality.

Carskadon, M. A., W. C. Dement	The multiple sleep latency test: What does it measure?	1982	Multiple sleep latency test measures physiological sleep tendency and provides an indication of individual difficulty maintaining arousal.

Caruso, C. C., E. M. Hitchcock	Strategies for nurses to prevent sleep-related injuries and errors	2010	The strategies to decrease sleep-related incidents include better sleep habits, better work schedules, napping, caffeine use, light exposure, and rest breaks.

Chasens, E. R., S. M. Sereika, L. E. Burke	Daytime sleepiness and functional outcomes in older adults with diabetes	2009	Among adults with diabetes aged 55 to 84 who reported excessive daytime sleepiness had higher BMI, lower subjective health, and more sleep disruptions.

Chen, Y., S. Li	The relationship between workplace ostracism and sleep quality: A mediated moderation model	2019	Workplace ostracism is associated with psychological detachment which decreases sleep quality. Humor can be used as a coping mechanism to decrease detachment and improve sleep.

Chung, S. A., T. K. Wolf, C. M. Shapiro	Sleep and health consequences of shift work in women	2009	Women shift workers experience poor sleep, take more sedatives, and have a wide range of negative health effects.

Cofer, L. F., J. W. Grice, L. Sethre-Hofstad, L. Radi	Developmental perspectives on morningness-eveningness and social interactions	1999	Individual variability in morning-eveningness is seen throughout development and remains stable into adulthood. Only dramatic environmental change will alter the chronotype.

Cohen, S., W. J. Doyle, C. M. Alper, D. Janicki-Deverts, R. B. Turner	Sleep habits and susceptibility to the common cold	2009	Sleep duration less than 7 hours was related to increased likelihood of developing a cold than sleep duration of 8 hours or more. Less than 92% sleep efficiency was related to increased likelihood of developing a cold than 98% efficiency or more.
Costa, G.	The impact of shift and night work on health	1996	Shift work negatively disturbs the natural circadian rhythms, decreases work efficiency, increases social problems, decreases health, and has specific negative effects on women.

Crowley, K.	Sleep and sleep disorders in older adults	2011	Sleep in older adults is often considered to be lighter or more fragile with decreased total sleep time, slow wave sleep, and REM as well as increased awakenings.

Czeisler, C. A., J. F. Duffy, T. L. Shanahan, E. N. Brown, et al.	Stability, precision, and near-24-hour period of the human circadian pacemaker	1999	Careful measures of the endogenous circadian rhythm of melatonin, core body temperature, and cortisol suggest that the internal circadian pacemaker in humans is 24.18 hours and has little variance.

Drake, C. L., T. Roehrs, G. Richardson, J. K. Walsh, T. Roth	Shift work sleep disorder: Prevalence and consequences beyond that of symptomatic day workers	2004	Approximately 10% of night and rotating shift workers experience shift work sleep disorder and are more likely to experience behavioral and health-related morbidity.

Dregan, A., D. Armstrong	Cross-country variation in sleep disturbance among working and older age groups: An analysis based on the European Social Survey	2011	Sleep habits and patterns varied across different European countries suggesting that sleep varies for different cultures. Older age groups generally reported more sleep disturbances than younger adults.

Driesen, K., N. W. Jansen, I. Kant, D. C. Mohren, L. G. van Amelsvoort	Depressed mood in the working population: Associations with work schedules and working hours	2010	Depressed mood occurred more for individuals working on shifts than those only working during the day with men being more affected than women. Men working fewer hours reported more depression while women working more hours reported more depression.

Duffy, J. F., S. W. Cain, A. M. Chang, A. J. Phillips, et al.	Sex difference in the near-24-hour intrinsic period of the human circadian timing system	2011	Women have shorter circadian periods than men which affects melatonin release and body temperature. Women also tend to wake up earlier and desire morning activities more than men.

Duffy, J. F., D. W. Rimmer, C. A. Czeisler	Association of intrinsic circadian period with morningness-eveningness, usual wake time, and circadian phase	2001	The endogenous circadian pacemaker is correlated with morningness – eveningness, circadian phase, and time awakening in the morning.

Empana, J. P., Y. Dauvilliers, J. F. Dartigues, K. Ritchie, et al.	Excessive daytime sleepiness is an independent risk indicator for cardiovascular mortality in community-dwelling elderly: The three city study	2009	Excessive daytime sleepiness is a risk factor for mortality in persons more than 64 years old even after adjusting for age, gender, study center (location), BMI, previous cardiovascular disease, mental state, and cardiovascular risk factors.

Engle-Friedman, M., S. Riela	Self-imposed sleep loss, sleepiness, effort and performance	2004	Although exam performance was not related to sleep time or sleepiness, decreased sleep time resulted in increased effort and concentration. Sleepiness predicted choosing to complete less difficult academic tasks.

Ertel, K. A., L. F. Berkman, O. M. Buxton	Socioeconomic status, occupational characteristics, and sleep duration in African/Caribbean immigrants and US white health care workers	2011	Minorities sleep less than white participants even when controlling for education, income, hours worked each week, and night shift work. Education, income, hours worked each week, and night shift work accounted for some of sleep duration disparity but not all.

Fortuna, L. R., B. Cook, M. V. Porche, Y. Wang, et al.	Sleep disturbance as a predictor of time to drug and alcohol use treatment in primary care	2018	Persons with sleep disturbances are more likely to have substance use problems.

Garbarino, S., O. Guglielmi, A. Sanna, G. L. Mancardi, N. Magnavita	Risk of occupational accidents in workers with obstructive sleep apnea: Systematic review and meta-analysis	2016	Workers with obstructive sleep apnea have nearly double the number of work accidents suggesting that screening for obstructive sleep apnea could be beneficial in many work settings.
Gelaye, B., V. Lohsoonthorn, S. Lertmeharit, W. C. Pensuksan et al.	Construct validity and factor structure of the Pittsburgh Sleep Quality Index and Epworth Sleepiness Scale in a multi-national study of African, south east Asian and South American college students	2014	Although both the Pittsburg Sleep Quality Index and Epworth Sleepiness Scale were developed as one-factor questionnaires, they showed multiple dimensions when examining both in a cross-cultural study.

Giannotti, F., F. Cortesi	Family and cultural influences on sleep development	2009	Family structure, cultural values and beliefs impact children’s sleep. There is no one perfect way to help children develop better sleep patterns.

Giorgi, G., G. Arcangeli, M. Perminiene, C. Lorini, et al.	Work-related stress in the banking sector: A review of incidence, correlated factors, and major consequences	2017	Stress is at critical levels in the banking industry. There are increases in mental health problems, such as anxiety and depression that resulted in maladaptive behaviors and job burnout.

Glance, D. J., E. Ooi, Y. Berman, Glance, C. F., H. R. Barrett	Impact of a digital activity tracker-based workplace activity program on health and wellbeing	2016	A workplace activity challenge was most effective with teams to provide social interaction and support. The challenge also resulted in decreases in non-HDL cholesterol and triglyceride concentrations, and improved health and wellbeing.

Glavas, A.	Corporate social responsibility and organizational psychology: An integrative review	2016	Corporate social responsibility can influence employees through self-engagement, self-interest, and morality by focusing on the human in the workplace not just productivity.

Grandner, M. A., N. J. Williams, K. L. Knutson, D. Roberts, G. Jean-Louis	Sleep disparity, race/ethnicity, and socioeconomic position	2016	Health disparities seen in racial or ethnic minorities could be due in part to differences in sleep where racial and ethnic minorities often report less or worse sleep.

Guest, D.	Human resource management, corporate performance and employee wellbeing: Building the worker into HRM	2002	Human resource management (HRM) has often focused on organizational performance and less on the workers. A most worker-friendly HRM could create a partnership context and improve mutual gains.

Hagger, M.S.	Where does sleep fit in models of self-control and health behaviour?	2014	Proposes a model integrating sleep sufficiency and consistency with self-control. The model is then extended to include the impact of self-control on health.

Harrington, J. M.	Health effects of shift work and extended hours of work	2001	Shift work and extended work hours are related to decreases in performance, problems with sleep, increased accident rates, increased mental health issues, and increased cardiovascular mortality rates.

Hirsch, A. J., J. E. Park, P. R. Daniele, J. Fleetham, et al.	Obstructive sleep apnoea and frequency of occupational injury	2016	Workers with obstructive sleep apnea are twice as likely to experience occupational injury. They are also almost three times more likely to suffer from occupational injury related to reduced vigilance.

Hoddes, E., V. Zarcone, H. Smythe, R. Phillips, W. C. Dement	Quantification of sleepiness: A new approach	1973	Reports on the development of the Stanford Sleepiness Scale, a single likert-type scale. Compared subjective sleepiness in subjects when rested and when sleep deprived.

Horne, J.A., O. Östberg	A self-assessment questionnaire to determine morning-eveningness in human circadian rhythms	1976	Describes the development of a morningness-eveningness scale and compares it to circadian variation in oral temperature. Morning types showed an earlier peak time in body temperature and a higher daytime body temperature than evening types.

Irwin, M. R.	Why sleep is important for health: A psychoneuroimmunology perspective	2015	Sleep affects the hypothalamic-pituitary-adrenal axis and the sympathetic nervous system which in turn regulate immune response. Sleep loss impairs adaptive immunity and increases inflammation.
Jaffe, M. P., M. H. Smolensky, C. C. Wun	Sleep quality and physical and social well-being in North American petrochemical shift workers	1996	Workers on an 8-hour backward shift system reported worse sleep quality, more gastrointestinal and cardiovascular complaints, and less family and personal time than workers on day shift or 12-hour shifts.

Jean-Louis, G., D. F. Kripke, S. Ancoli-Israel, M. R. Klauber, R. S. Sepulveda	Sleep duration, illumination, and activity patterns in a population sample: Effects of gender and ethnicity	2000	Actigraphic data suggest that adults sleep less than 6.5 hours each night and that sleep duration gradually decreases in older adults. Women slept more than men and minorities (especially men) slept less. Men and women had similar activity levels but men were exposed to brighter illumination.

Jenni, O. G., B. B. O’Connor	Children’s sleep: An interplay between culture and biology	2005	Cultural environments, beliefs, and values impact children’s sleep patterns.

Johns, M. W.	A new method for measuring daytime sleepiness: The Epworth Sleepiness Scale	1991	Describes development of the Epworth Sleepiness Scale which measures sleepiness by estimating the chance of falling asleep during eight common situations.

Jones, C. B., J. Dorrian, S. M. Jay, N. Lamond, S. Ferguson, D. Dawson	Self-awareness of impairment and the decision to drive after an extended period of wakefulness	2006	Self-assessment of potential driving skills mirrored decrements in performance on the PVT when sleep deprived for 40 hours. However, rated others as less capable of driving under similar conditions.

Kato, M., B. G. Phillips, G. Sigurdsson, K. Narkiewicz, et al.	Effects of sleep deprivation on neural circulatory control	2000	Sleep deprivation increases resting blood pressure and decreases muscle sympathetic nerve activity.

Kecklund, G., J. Axelsson	Health consequences of shift work and insufficient sleep	2016	Reviews the impact of shift work, particularly night shift and early morning shifts, on sleep loss and the link with increased health risk including heart disease, diabetes, stroke, and cancer.

Knez, I.	Toward a model of work-related self: A narrative review	2016	Work-related identity is a complex structure including organizational, workgroup and professional identity, each with their own impact on the individual.

Knutsson, A.	Health disorders of shift workers	2003	Shift work and night shifts are related to increase incident rates of peptic ulcer disease, coronary heart disease, and problems with preganancies.

Kolla, B. P., S. Mansukhani, M. P. Mansukhani	Consumer sleep tracking devices: A review of mechanisms, validity and utility	2016	Sleep tracking devices tend to overestimate sleep times and underestimate sleep efficiency.

Kripke, D. F., L. Garfinkel, D. L. Wingard, M. R. Klauber, M. R. Marler	Mortality associated with sleep duration and insomnia	2002	Highest survival rates were found for those reporting 7 hours sleep/night. There was an increased mortality risk for those reporting more than 8.5 or less than 4.5 hours sleep/night.

Krueger, P. M., E. M. Friedman	Sleep duration in the United States: A cross-sectional population-based study	2009	Sleep duration is related to multiple demographic, family structure, socioeconomic, health behavior, and health status constructs.

Kucharczyk, E. R., K. Morgan, A. P. Hall	The occupational impact of sleep quality and insomnia symptoms	2012	Insomnia negatively affects workplace absenteeism, accident risk, productivity, career progression, and job satisfaction.

Lan, L., K. Tsuzuki, Y. Liu, Z. Lian	Thermal environment and sleep quality: A review	2017	Cold air temperatures can negatively impact sleep, especially REM sleep. Warm air temperatures can be countered through proper air flow.

Lange, T., S. Dimitrov, J. Born	Effects of sleep and circadian rhythm on the human immune system	2010	The circadian system and sleep influence neuroendocrine mechanisms and control over immune functioning.
Lansdall-Welfare, T., S. Lightman, N. Cristianini	Seasonal variation in antidepressant prescriptions, environmental light and web queries for seasonal affective disorder	2019	Prevalence of seasonal affective disorder can be monitored through web queries. Day length is a better correlate of seasonal affective disorder than environmental bright light exposure.

Lim, J., D. F. Dinges	A meta-analysis of the impact of short-term sleep deprivation on cognitive variables	2010	Short-term total sleep deprivation has differential effects on cognitive processes with attention being most negatively affected.

Litwiller, B., L. A. Snyder, W. D. Taylor, L. M. Steele	The relationship between sleep and work: A meta-analysis	2017	Sleep quality and sleep quantity differ in terms of their relationships to subjective measures related to health, affect, and attitudes.

Lopez, R., I. Jaussent, S. Scholz, S. Bayard, J. Montplaisir, Y. Dauvilliers	Functional impairment in adult sleepwalkers: A case-control study	2013	Adult sleepwalking negatively impacts health and quality of life through violent behaviors and sleep disruption.

Luckhaupt, S. E., S. Tak, G. M. Calvert	The prevalence of short sleep duration by industry and occupation in the national health interview survey	2010	Short sleep duration has increased in many occupations. Greater levels of sleepiness in management, transportation and manufacturing.

Maide, J. A., J. M. Krueger	Links between the innate immune system and sleep	2005	Sleep is mediated by cytokines, an immune mediator. Sleep, in turn, helps maintain effective immune functioning.

Mariappanadar, S.	Stakeholder harm index: A framework to review work intensification from the critical HRM perspective	2014	Proposes a stakeholder harm index with a focus on high performance work practices (HPWP). HPWP can cause psychological, social, and work-related health problems.

Marquie, J., J. Foret	Sleep, age, and shiftwork experience	1999	Current and previous shift workers experience more sleep-related problems. However, the length or recency of shift work had no effect. Women were more negatively affected by shift work with age.

McClelland, L. E., F. S. Switzer III, J. J. Pilcher	Changes in nurses’ decision making during a 12-h day shift	2013	Judgements on a task delineating medical-based scenarios changed across a 12-hour work shift in nurses suggesting the use of different decision criteria over the work shift.

McCubbin, J. A., J. J. Pilcher, D. D. Moore	Blood pressure increases during a simulated night shift in persons at risk for hypertension	2010	Throughout a night of sleep deprivation, prehypertensive participants had elevated blood pressure, while participants with a positive family history of hypertension but current normal blood pressure levels had higher resting diastolic blood pressure.

Mecacci, L., G. Rocchetti	Morning and evening types: Stress-related personality aspects	1998	Eveningness is more related to neuroticism, psychoticism, and anxiety than morningness. Eveningness is also related to difficulty sustaining effort in stimulating conditions and adapting to changing environmental demands.

Meier-Ewert, H. K., P. M. Ridker, N. Rifai, M. M. Regan, et al.	Effect of sleep loss on C-reactive protein, an inflammatory marker of cardiovascular risk	2004	C-reactive proteins a marker of inflammation, increased during total and partial sleep deprivation.

Melamed, S., A. Oksenberg	Excessive daytime sleepiness and risk of occupational injuries in non-shift daytime workers	2002	Excessive daytime sleepiness is common in daytime workers and is related to increased risk of on-the-job injury. Informing workers of this relationship helps decrease injuries.

Miller, G. E., S. Cohen, S. Pressman, A. Barkin, et al.	Psychological stress and antibody response to influenza vaccination: When is the critical period for stress, and how does it get inside the body?	2004	Increased levels of self-reported stress were related to poorer antibody response to a flu vaccine for a 10 day period after the vaccination. Feelings of stress and sleep loss diminished the immune response.

Mitler, M. M., K. S. Gujavarty, C. P. Browman	Maintenance of wakefulness test: A polysomnographic technique for evaluating treatment efficacy in patients with excessive somnolence	1982	Maintenance of wakefulness test measures length of time to sleep onset when individual is sitting comfortably and trying to stay awake.
Mongrain, V., J. Carrier, M. Dumont	Circadian and homeostatic sleep regulation in morningness-eveningness	2006	Chronotype may develop in response to specific sleep EEG patterns that affect the dissipation of sleep pressure independent of circadian processes that encourage sleep.

Monk, T. H., D. J. Kupfer	Which aspects of morningness-eveningness change with age?	2007	Morning-eveningness scores suggest that people become more morning types as they age. Older individuals report greater morning alertness with a resulting inability to sleep late than younger individuals.

Moore, P. J., N. E. Adler, D. R. Williams, J. S. Jackson	Socioeconomic status and health: The role of sleep	2002	Sleep quality mediates the effect of income on mental and physical health. Sleep quantity was related to mental and physical health but not socioeconomic status.

Morris, D. M., J. J. Pilcher, J. B. Mulvihill, M. A. Vander Wood	Performance awareness: Predicting cognitive performance during simulated shiftwork using chronobiological measures	2017	Changes in oral temperature and heart rate are predictive of performance awareness under simulated nightshift conditions. Oral temperature alone also significantly predicts performance awareness.

Morris, D. M., J. J. Pilcher, R. B. Powell	Task-dependent cold stress during expeditions in Antarctic environments	2017	Cold stress differentially impacts occupational performance and may moderate the risk for hypothermia.

Morris, D. M., J. J. Pilcher, F. S. Switzer III	Lane heading difference: An innovative model for drowsy driving detection using retrospective analysis around curves	2015	Calculating changes in lane heading provides a better model for detecting drowsy driving than other commonly used metrics.

Mullins, H. M., J. M. Cortina, C. L. Drake, R. S. Dalal	Sleepiness at work: A review and framework of how the physiology of sleepiness impacts the workplace	2014	Sleepiness has major consequences for organizations and employees including decreased performance, changes in affect and emotional reaction, increased accidents, and increased deviant behaviors.

Mysliwiec, V., B. O’Reilly, J. Polchinski, H. P. Kwon, et al.	Trauma associated sleep disorder: A proposed parasomnia encompassing disruptive nocturnal behaviors, nightmares, and REM without atonia in trauma survivors	2014	Following a traumatic experience, persons had disruptive nocturnal behaviors, nightmares replaying the event, and a lack of atonia during REM.

Nakamura, T. J., W. Nakamura, S. Yamazaki, T. Kudo, et al.	Age-related decline in circadian output	2011	In aging mice, there is less activity in the suprachiasmatic nucleus and may precede disruption of the molecular circadian rhythms.

Nakata, A., T. Haratani, M. Takahashi, N. Kawakami, et al.	Job stress, social support, and prevalence of insomnia in a population of Japanese daytime workers	2004	Increased insomnia is related to increased levels of intergroup conflict, job dissatisfaction, and depression and more weakly associated with poor employment opportunities, physical environment, and coworker support.

Ng, T. W., D. C. Feldman	The relationships of age with job attitudes: A meta-analysis	2010	Older workers tend to have more positive job attitudes than younger workers. Older workers also report higher levels of motivation and job involvement with lower levels of job depersonalization than younger workers. Older workers report higher satisfaction, loyalty, and affective commitment than younger workers.

Odle-Dusseau, H. N., J. L. Bradley, J. J. Pilcher	Subjective perceptions of the effects of sustained performance under sleep-deprivation conditions	2010	Sleep deprived persons reported increased effort on cognitive and vigilance tasks, but effort varied across the night of sleep deprivation. Subjective motivation was higher for cognitive tasks but decreased for both tasks across the night. Perceived stress did not change across the night.

Ohayon, M. M., M. A. Carskadon, C. Guilleminault, M. V. Vitiello	Meta-analysis of quantitative sleep parameters from childhood to old age in healthy individuals: Developing normative sleep values across the human lifespan	2004	Sleep latency, stage 1, and stage 2 increase with age in adults. REM decreases with age in adults.
Pagel, J.	Excessive daytime sleepiness	2009	Causes of excessive daytime sleepiness include sleep deprivation, sedating medications, and some sleep disorders. Obstructive sleep apnea is a common cause of daytime sleepiness.

Paschos, G. K., G. A. FitzGerald	Circadian clocks and metabolism: Implications for microbiome and aging	2017	The endogenous circadian clock regulates cellular energy and cellular energy regulated the circadian clock. The alignment of the central clock and the peripheral clocks could have an impact on disease and aging.

Pereira, D., L. L. Meier, A. Elfering	Short-term effects of social exclusion at work and worries on sleep	2013	Social exclusion in the workplace and work-related worries are related to increased sleep fragmentation.

Philip, P., J. Taillard, I. Niedhammer, C. Guilleminault, B. Bioulac	Is there a link between subjective daytime somnolence and sickness absenteeism? A study in a working population	2001	Even after adjusting for age, sex, employment grade, sleep symptoms, and self-reported diseases, there is a strong association between subjective daytime somnolence and absence due to sickness.

Pilcher, J. J., D. Band, H. N. Odle-Dusseau, E. R. Muth	Human performance under sustained operations and acute sleep deprivation conditions: Toward a model of controlled attention	2007	Impact of sleep deprivation depends on type of task where tasks that require controlled attention (e.g., vigilance tasks) are more negatively affected by sleep deprivation than more intrinsically interesting tasks.

Pilcher, J. J., S. A. Bryant	Implications of social support as a self-control resource	2016	Social support may help reduce stress and contribute to self-control.

Pilcher, J. J., C. Callan, J. L. Posey	Sleep deprivation affects reactivity to positive but not negative stimuli	2015	Partial and total sleep deprivation negatively affect reactions to positive more than negative emotional stimuli. Negative events could be resistant to the effects of sleep deprivation because they more readily elicit attention from sleep-deprived persons.

Pilcher, J. J., D. R. Ginter, B. Sadowsky	Sleep quality versus sleep quantity: Relationships between sleep and measures of health, well-being and sleepiness in college students	1997	Sleep quality is better related to health, well-being, and sleepiness than sleep quantity when the person is sleeping an average of 7 hours a night across a 7-day period.

Pilcher, J. J., A. I. Huffcutt	Effects of sleep deprivation on performance: A meta-analysis	1996	Total and partial sleep deprivation impairs performance. Mood is more affected by total and partial sleep deprivation than cognitive or motor performance.

Pilcher, J. J., K. S. Jennings, G. E. Phillips, J. A. McCubbin	Auditory attention and comprehension during a simulated night shift: Effects of task characteristics	2016	Simulated night shift has a greater negative effect on auditory material that is less interesting and more difficult.

Pilcher, J. J., B. J. Lambert, A. I. Huffcutt	Differential effects of permanent and rotating shifts on self-report sleep length: A meta-analytic review	2000	Slowly rotating shifts have the least negative impact on sleep length. In work settings requiring frequent night shifts, permanent night shifts can be less problematic than rotating shifts.

Pilcher, J. J., D. M. Morris, J. Donnelly, H. B. Feigl	Interactions between sleep habits and self-control	2015	Sleep deprivation can lead to uncontrolled impulses, problems with attentional control, and poor decision making.

Pilcher, J. J., H. Schulz	The interaction between EEG and transient muscle activity during sleep in humans	1987	Transient muscle activity occurs more frequently during desynchronized brain activity. Synchronized brain activity is associated with less transient muscle activity.

Pilcher, J. J., F. S. Switzer III, A. Munc, J. Donnelly, J. C. Jellen, C. Lamm	Psychometric properties of the Epworth Sleepiness Scale: A factor analysis and item-response theory approach	2018	The Epworth Sleepiness Scale measures two constructs: active and passive responding. Item 8 on the scale and Item 6 but less so (both portray more active situations) are interpreted differently than the remaining items.

Pitkanen, M., J. Hurn, M. D. Kopelman	Doctors’ health and fitness to practice: Performance problems in doctors and cognitive impairments	2008	Focuses on medical doctors’ showing a sustained pattern of performance decrements and considers potential impact of neuropsychiatric and neuropsychological disorders.

Preckel, F., A. A. Lipnevich, S. Schneider, R. D. Roberts	Chronotype, cognitive abilities, and academic achievement: A meta-analytic investigation	2011	Morningness is negatively related to cognitive ability but positively related to academic achievement while eveningness is positively related to cognitive ability but negatively related to academic achievement.

Ramin, C., E. E. Devore, W. Wang, J. Pierre-Paul, et al.	Night shift work at specific age ranges and chronic disease risk factors	2015	Night shift workers experience greater chances of obesity, more caffeine intake, higher calorie intake, increased smoking rates, and shorter sleep duration, particularly in shift workers older than 25.

Randler, C.	Morningness-eveningness comparison in adolescents from different countries around the world	2008	Chronotype is influenced by climate, longitude and latitude. Temperate climates are associated with eveningness whereas morningness is associated with more northern and eastern locales.

Randler, C., P. Prokop, S. Sahu, P. Haldar	Cross-cultural comparison of seven morningness and sleep-wake measures from Germany, India and Slovakia	2015	Morningness-eveningness varied across countries with Germans being the latest chronotypes.

Roenneberg, T., T. Kuehnle, M. Juda, T. Kantermann, et al.	Epidemiology of the human circadian clock	2007	A simple questionnaire can assess human chronotype. The chronotype depends on age, sex, and light exposure.

Rongen, A., S. J. Robroek, F. J. van Lenthe, A. Burdorf	Workplace health promotion: A meta-analysis of effectiveness	2013	Workplace health promotion has limited effect but is larger with younger people with weekly contacts and when compared to a control group of no health promotion.

Rosekind, M. R., K. B. Gregory, M. M. Mallis, S. L. Brandt, et al.	The cost of poor sleep: Workplace productivity loss and associated costs	2010	Insomnia and insufficient sleep leads to decreased work productivity, performance, and safety. Persons with insomnia were more likely to use sleep medications.

Rosenthal, N. E., D. A. Sack, J. C. Gillin, A. J. Lewy, et al.	Seasonal affective disorder: A description of the syndrome and preliminary findings with light therapy	1984	Persons suffering with seasonal affective disorder experience depressive symptoms depending on changes in the seasonal exposure to sunlight. Using early-morning light therapy to extend light exposure may have antidepressant effects.

Ruiter, M. E., J. DeCoster, L. Jacobs, K. L. Lichstein	Normal sleep in African-Americans and Caucasian-Americans: A meta-analysis	2011	African-Americans experience worse sleep continuity and less sleep duration as assessed by objective and subjective measures.

Shahid, A., J. Shen, C. M. Shapiro	Measurements of sleepiness and fatigue	2010	Discusses the theoretical differences between sleepiness and fatigue and need for better distinction between the two. Describes measures of sleepiness and fatigue including subjective and objective measures.

Smolders, K. C., Y. A. de Kort	Bright light and mental fatigue: Effects on alertness, vitality, performance and physiological arousal	2014	Participants reported being less sleepy, more vital, and happier after bright light exposure. When combined with mental fatigue, participants reported less sleepiness and better self-control. Effects on performance was mixed.

Spiegel, K., R. Leproult, E. Van Cauter	Impact of sleep debt on metabolic and endocrine function	1999	Sleep loss lowered glucose tolerance and thyrotropin levels and increased evening cortisol levels and sympathetic nervous system activity.

Spiegel, K., J. F. Sheridan, E. Van Cauter	Effect of sleep deprivation on response to immunization	2002	Sleep deprivation decreased the antibody response to vaccination.

Stranges, S., W. Tigbe, F. X. Gómez-Olivé, Thorogood, M., N. B. Kandala	Sleep problems: An emerging global epidemic? Findings from the INDEPTH WHO-SAGE study among more than 40,000 older adults from 8 countries across Africa and Asia	2012	Reported rates of sleep difficulties differed across countries with higher rates of sleep difficulty in women and in older age groups. Increased sleep difficulties were also related to lower education levels, not living with a partner, poorer quality of life, and feelings of depression and anxiety.
Suzuki, K., T. Ohida, Y. Kaneita, E. Yokoyama, M. Uchiyama	Daytime sleepiness, sleep habits and occupational accidents among hospital nurses	2005	Excessive daytime sleepiness in nurses was related to increased occupational accidents such as drug administration errors and difficulty correctly using medical equipment.

Swanson, L. M., J. Arnedt, M. R. Rosekind, G. Belenky, et al.	Sleep disorders and work performance: Findings from the 2008 National Sleep Foundation Sleep in America poll	2011	Long work hours are associated with less sleep and less sleep is associated with increase problems at work. Individuals with sleep disorders (37% of respondants) reported more problems at work and greater work absenteeism.

Tochikubo, O., A. Ikeda, E. Miyaiima, M. Ishii	Effects of insufficient sleep on blood pressure monitored by a new multibiomedical recorder	1996	Blood pressure, the low to high frequency ratio of the RR interval, and heart rate were increased during the day following a night shift.

Touitou, Y., A. Reinberg, D. Touitou	Association between light at night, melatonin secretion, sleep deprivation, and the internal clock: Health impacts and mechanisms of circadian disruption	2017	Working at night disrupts the circadian cycle and sleep. Exposure to artificial light at night amplifies these problems and could contribute to health-related problems associated with shiftwork by interfering with the natural functioning of the endocrine system.

Uehli, K., A. J. Mehta, D. Miedinger, K. Hug, et al.	Sleep problems and work injuries: A systematic review and meta-analysis	2014	Workers who report sleep difficulties have a higher risk of being injured on the job than workers without sleep difficulties.

Urtasun, A., I. Nuñez	Healthy working days: The (positive) effect of work effort on occupational health from a human capital approach	2018	In adequate working conditions, health remains stable up through 120 h/week of work, while in inadequate working conditions, health can start to deteriorate with only 35 h/week of work.

van der Helm, E., N, Gujar, M. P. Walker	Sleep deprivation impairs the accurate recognition of human emotions	2010	Sleep deprivation negatively impacts judgment of facial emotion particularly threat or anger and reward or happy emotions.

Van Dongen, H. P.	Shift work and inter-individual differences in sleep and sleepiness	2006	Individuals react differently to shift work with person-specific differences in performance and alertness.

Vgontzas, A. N., E. Zoumakis, E. Bixler, O., H. Lin, et al.	Adverse effects of modest sleep restriction on sleepiness, performance, and inflammatory cytokines	2004	Sleep restriction to 6 hours a night for one week increased objective sleepiness, decreased vigilance performance, and increased IL-6 (inflammatory cytokines) secretion.

Voronov, M., R. Vince	Integrating emotions into the analysis of institutional work	2012	Investment and disinvestment are important components of understanding institutional work.

Walker, A. D., E. R. Muth, H. N. Odle-Dusseau, D. W. Moore, J. J. Pilcher	The effects of 28 hours of sleep deprivation on respiratory sinus arrhythmia during tasks with low and high controlled attention demands	2009	Respiratory sinus arrhythmia follows a circadian pattern for low controlled attention tasks but not high controlled attention tasks, suggesting that parasympathetic activity and task type affect task performance under sleep deprivation conditions.

Watanabe, T., K. Watanabe	Noncontact method for sleep stage estimation	2004	Reports on a pneumatic method for scoring sleep stages, as a less invasive method to monitor sleep.

Wong, M. M., K. J. Brower, H. E. Fitzgerald, R. A. Zucker	Sleep problems in early childhood and early onset of alcohol and other drug use in adolescence	2004	Mother ratings of sleep problems in early childhood predict early onset of alcohol, marijuana, and other drug use in early adolescence as well as attention problems and anxiety/depression.

Shift work scheduling, travel across multiple time zones, and working in the circumpolar regions contribute to on-the-job sleepiness, circadian disruption, and decreased task performance. Best practices for organizational intervention to mitigate these work-related hazards require additional research. At least some of this difficulty comes from the complex interaction between employee individual differences and differences in occupations (Van Dongen, 2006). In addition, the choice by the worker on whether to prioritize sleep has an impact. Many workers are willing to exchange personal sleep time to increase time at work and time with their family (Barnes et al., 2012). Although organizations cannot control each employee’s sleep times, human resource management can investigate more thorough education programs as well as potential actions in the workplace that would help workers maintain better sleep habits and alertness when on-the-job. Personnel scheduling and responsibilities, demographics, and personal traits contribute to sleep habits and the risk of sleepiness in the workplace as well as a decrease in occupational-related performance. However, the degree to which this on-the-job sleepiness limits job performance varies depending on individual tolerances to job demands as well as the job characteristics. As such, implementing best practices and interventions in at-risk occupations is a challenging but necessary process for workers, human resources, and organizations.

Current research supports introducing workplace programs to support healthy sleep habits. Some organizations may assume that employees will make responsible and rational decisions about their sleep health. However, without education, it is difficult for many people to fully appreciate how many aspects of performance and health are negatively impacted by poor sleep habits and sleep loss. Although the effectiveness of workplace health promotion programs depends on the type of intervention and characteristics of the workers (Rongen et al., 2013), it is essential to develop and test health promotion programs that address specific occupational concerns. For example, health promotion programs that utilize wearable technology, such as creating teams to use step counters to track movement, show greater participation and less attrition than with individual participants (Glance et al., 2016). Health promotion programs could provide a range of effort such as, educational materials, classes, and health-promotion events that could help inform employees of the importance of prioritizing sleep as well as indicating the organization’s commitment to helping the employees. The programs could also focus on using current technology to better track and improve sleep habits and alertness when working. Better developing and maintaining health promotion programs such as these could benefit the employees as well as the organization.

## Author Contributions

Both authors contributed in the conceptual development of the manuscript and writing the manuscript.

## Conflict of Interest

The authors declare that the research was conducted in the absence of any commercial or financial relationships that could be construed as a potential conflict of interest.
